# Neuroinflammation and the Gut Microbiota: Possible Alternative Therapeutic Targets to Counteract Alzheimer’s Disease?

**DOI:** 10.3389/fnagi.2019.00284

**Published:** 2019-10-18

**Authors:** Milica Cerovic, Gianluigi Forloni, Claudia Balducci

**Affiliations:** Department of Neuroscience, Istituto di Ricerche Farmacologiche Mario Negri, IRCCS, Milan, Italy

**Keywords:** Alzhimer’s disease, neuroinflammation, gut microbiota, immune cells, therapy

## Abstract

Alzheimer’s disease (AD) is a complex, multi-factorial disease affecting various brain systems. This complexity implies that successful therapies must be directed against several core neuropathological targets rather than single ones. The scientific community has made great efforts to identify the right AD targets beside the historic amyloid-β (Aβ). Neuroinflammation is re-emerging as determinant in the neuropathological process of AD. A new theory, still in its infancy, highlights the role of gut microbiota (GM) in the control of brain development, but also in the onset and progression of neurodegenerative diseases. Bidirectional communication between the central and the enteric nervous systems, called gut-brain axes, is largely influenced by GM and the immune system is a potential key mediator of this interaction. Growing evidence points to the role of GM in the maturation and activation of host microglia and peripheral immune cells. Several recent studies have found abnormalities in GM (dysbiosis) in AD populations. These observations raise the intriguing question whether and how GM dysbiosis could contribute to AD development through action on the immune system and whether, in a therapeutic prospective, the development of strategies preserving a healthy GM might become a valuable approach to prevent AD. Here, we review the evidence from animal models and humans of the role of GM in neuroinflammation and AD.

## Introduction

Alzheimer’s disease (AD) remains the most spread form of dementia afflicting 45 million people worldwide and continuously challenging the scientific community in the hard task of identifying a therapy. AD pathogenesis starts around 15–20 years before the clinical symptoms become detectable. Within this time frame, the patient’s brain accumulates multiple-system damages including synaptic and mitochondrial alterations, vessel damage, chronic neuroinflammation, cognitive dysfunctions and neuronal cell death. amyloid-β (Aβ) extracellular plaques and intracellular neurofibrillary tangles enriched of hyperphosphorylated Tau protein are the two main histological brain lesions. Aβ is still held to be the main culprit, especially in its soluble oligomeric form. Aβ oligomers are indeed considered as the most neurotoxic species and the best correlates of disease severity (Forloni et al., 2016). However, Aβ can no longer be considered the sole target because of the multiple failures in anti-Aβ trials (Panza et al., 2019). More likely, the complex pathological condition of AD conceivably calls for alternative targets and multi-target therapies.

Based on these considerations, this review aims to highlight two therapeutic targets, which are attracting much attention in the fight against AD: neuroinflammation and the gut microbiota (GM). The former has strongly re-emerged as crucial in the neuropathogenic process of AD, whereas the latter, though still in its infancy, is attracting interest as a promising new alternative target. Both systems intimately interact in physiology and pathology.

## Neuroinflammation and AD

A large body of evidence has accumulated in the last few years on the vital role of neuroinflammation in the pathogenetic process of AD. In physiological conditions glial cells are determinants in the regulation of brain development, neuronal activity and survival. Microglia patrol the brain microenvironment guaranteeing its defense from exogenous pathogens or endogenous dangers. In response to bacterial and viral infections or brain damage, microglia are rapidly activated and phagocytize pathogens, including Aβ, and damaged neurons. With elimination of harmful stimulus, neuroinflammation is resolved and microglia return to a resting state. In AD, neuroinflammation is chronic and resolution is not achieved. This implies that microglia constantly release pro-inflammatory cytokines, favoring neuronal cell death (Heneka et al., 2015). Indeed, many of the inflammatory mediators, such as pro-inflammatory cytokines, chemokines as well as factors of the complement system are produced locally and elevated in the brain of AD patients (Rogers et al., 1992; McGeer and McGeer, 2002). The most representative cytokines of AD are IL1β, TNFα and IL6, all up-regulated in AD tissues and prominently associated with AD lesions (Griffin et al., 1989; Dickson et al., 1993). It was recently demonstrated that neurodegeneration very likely involves astrocytes which, by taking on a microglia-induced A1 pro-inflammatory phenotype, would promote neuronal cell death, with TNFα as the most prominent mediator (Fiala and Veerhuis, 2010; Liddelow et al., 2017). In addition, activated microglia loses their phagocytic properties, thus reducing the degree of Aβ phagocytosis, and consequently promoting its accumulation (Krabbe et al., 2013). These findings are supported by the discovery of a relation between an increase in AD risk and mutations in genes encoding immune receptors such as TREM2, myeloid cell surface antigen CD33 and CR1 (Balducci and Forloni, 2018). This was compelling since they are all expressed on myeloid cells, thus demonstrating that alterations in microglial biology are linked to AD pathogenesis and an increased risk of its development. Of note, a series of transcriptomic and proteomic analysis of inflammatory cells might provide biomarkers for preclinical detection as well as insights on progression from MCI to AD condition (Fiala and Veerhuis, 2010; Wes et al., 2016; Rangaraju et al., 2018; Bonham et al., 2019).

A close relation has also been described between primed microglia and cognitive dysfunction. In healthy tissue, microglia have a ramified morphology and the prolongations constantly survey synaptic activity. Phagocytic microglia have an important role in synaptic pruning and refinement in the developing nervous system (Weinhard et al., 2018). The most intriguing mechanism explaining memory dysfunction in AD implies that Aβ oligomers, the most toxic species, foster microglial activation which then excessively engulf and eliminate synapses through C1q and C3 complement factors (Hong et al., 2016). We too have reported that Aβ oligomer-mediated memory impairment is closely associated with glial activation (Balducci et al., 2017).

New evidence is now shedding light on a dangerous dialogue between central immune cells and the gut, potentially leading to AD.

## Microbiota-Gut-Brain Axes

Constant communication between the central and enteric nervous systems is required to maintain body homeostasis. This complex interplay, the “Gut-brain-axis,” is mediated by neural, endocrine and immune signals (Carabotti et al., 2015). GM, the dense population of bacteria, viruses, fungi, and protozoa inhabiting the human gut, is now recognized as an important part of this interaction and the new term Microbiota-Gut-Brain axis, has been introduced. Recent progress in high-throughput analyses has permitted to study more in-depth the microbial composition and appreciate its complexity (Rooks and Garrett, 2016).

Every person has a distinct and widely variable GM, with some common features emerging only at higher level of organization (Tremaroli and Bäckhed, 2012). This dynamic system is subject to re-modeling in response to aging, environmental perturbations, changes in lifestyle and diet and it is therefore prone to maladaptive modifications (Santoro et al., 2018). Substantial shifts in human GM composition have been observed in CNS disorders such as depression, anxiety, autism (Finegold et al., 2012; Liang et al., 2018) and neurodegeneration (Fung et al., 2017).

## Gut Microbiota in CNS Physiology

Germ free (GF) and antibiotic-treated rodents provided the necessary tool to study the impact of intestinal microbes on CNS development and physiology.

A pioneering study used GF mice, which are generated and raised in sterile conditions, to investigate the influence of GM on hypothalamic-pituitary-adrenal (HPA) response to stress. The HPA response was significantly higher in GF relative to mice raised with normal GM. The introduction of the complex microbiota at an early stage (up to 9 weeks old), could partially reverse this enhanced HPA response to stress. GF mice also had lower brain-derived neurotrophic factor (BDNF) expression, which is important for neuronal growth and synaptic plasticity, in the cortex and hippocampus (Sudo et al., 2004).

Subsequent studies showed that the absence of complex microbiota has profound effects on adult behavior and CNS development and that the timing and duration of exposure to microorganisms are critical. GF condition altered spatial, working and reference memory, increased motor activity and reduced anxiety (Diaz Heijtz et al., 2011; Gareau et al., 2011). It also impaired hippocampal development and morphology, increased dorsal hippocampal neurogenesis and BBB permeability, and significantly altered levels of noradrenaline, dopamine and serotonin (Braniste et al., 2014; Luczynski et al., 2016; Sharon et al., 2016; Lin et al., 2018).

## Symbiotic Relationship Between the Immune System and GM

The microbial ecosystem co-evolved with our immune system over millennia and host-specific antimicrobial peptides and pattern recognition receptors evolved not only to protect against pathogens but also to promote resident beneficial microbes (Bosch, 2014). The immune system closely controls the GM composition and distribution (Sigal and Meyer, 2016), while the microbial symbionts regulate immune system maturation and function (Belkaid and Hand, 2014). Commensal GM can profoundly affect both innate and adaptive immune systems. Several studies have confirmed the interaction between microbiota and various immune cell populations: peripheral T cells, myeloid cells and mast cells (Round and Mazmanian, 2009; Kamada et al., 2013; Forsythe, 2016).

Khosravi et al. (2014) provided evidence that GM influences the development of the immune system by regulating hematopoiesis of primary immune cells. They showed that GF mice have lower proportions and less differentiation potential of myeloid cell progenitors of both yolk sac and bone marrow origin. This could help explain the widespread effects of GM on the immune system, microglia included.

## GM-Microglia Interaction

Mounting evidence from animal studies demonstrates that GM regulates microglial maturation and function. Microglia from GF or antibiotic-treated mice had an immature profile and impaired immune response (Erny et al., 2015). The absence of gut flora altered microglia mRNA profiles and downregulated several microglial genes involved in cell activation, pathogen recognition and host defense. The microglia transcription and survival factors SFPI1 and CSF1R, normally downregulated in mature adult microglia, were upregulated in GF mice (Kierdorf and Prinz, 2013). Matcovitch-Natan et al. (2016) examined the transcriptional profiles of different microglial development stages, showing that the genes related to the adult phase of microglial maturation and immune response are dysregulated in GF mice.

Products derived from bacterial metabolism such as short-chain fatty acids (SCFAs) were identified as key mediators of GM-microglia interaction. These molecules are able to translocate from colonic mucosa to systemic circulation (Schönfeld and Wojtczak, 2016), cross the BBB and affect CNS function (Borre et al., 2014). A SCFA supplement in drinking water of GF mice for 4 weeks restored many aspects of the immature microglial morphology, re-established microglial density and normalized CSF1R surface expression (Erny et al., 2015).

From the therapeutic perspective, it is important to highlight that the GM-microglia interaction is highly dynamic as many of the defects observed in microglia of GF mice could be partially restored by recolonization with conventional GM or SCFA supplementation.

## GM Alterations in AD

An association between gut dysbiosis and neurodegeneration is mostly supported by pre-clinical studies, while the clinical data are still limited. The most consistent clinical evidence of deviation from the healthy microbial composition in a neurodegenerative condition derives from studies of Parkinson’s disease (PD) patients (Keshavarzian et al., 2015; Scheperjans et al., 2015; Hopfner et al., 2017). Only few studies have investigated GM populations in AD patients. Microbial diversity was reduced in AD patient feces compared to age- and sex-matched controls. At the phylum level, there was a decrease in the numbers of Firmicutes and Actinobacteria and an increase in abundance of Bacteroidetes. The relative abundance of bacterial genera correlated with the levels of cerebrospinal fluid biomarkers of AD (Vogt et al., 2017). A recent study examined the link between selected bacterial taxa and brain amyloidosis in patients with cognitive impairment. Amyloid deposition was associated with an increased stool content of the pro-inflammatory taxa *Escherichia/Shigella* and low anti-inflammatory taxon *Eubacterium rectale*. These changes correlated well with a peripheral inflammatory state (Cattaneo et al., 2017). A few human studies have also linked dysbioses of oro-nasal cavity microbiota with neurodegeneration (Kamer et al., 2008; Cockburn et al., 2012).

**Figure 1:**
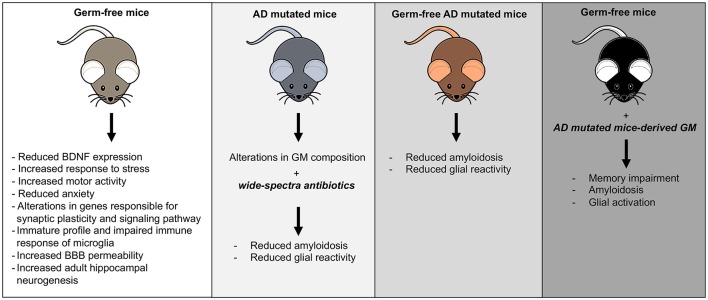
Mouse models of gut microbiota (GM) manipulation. The figure illustrates the typical mouse phenotypes resulting from various GM manipulations.

In AD animal models was found a significant shift in the composition of GM, and microbial manipulations could affect disease outcomes as summarized in Figure 1 (Brandscheid et al., 2017; Harach et al., 2017; Shen et al., 2017). A combination of broad-spectrum antibiotics or GF condition in AD mice reduced Aβ plaques and attenuated plaque-surrounding glial reactivity and the levels of circulating cytokines and chemokines (Minter et al., 2016). Conversely, re-introduction of conventionally raised AD mice gut flora in GF mice increased Aβ pathology (Harach et al., 2017).

## How Changes in Microbial Composition Could Be Relevant to AD

A plethora of hypotheses have been advanced to explain possible mechanisms linking GM alteration to neurodegenerative processes, many of them involving neuroinflammation as a common driving force.

The GM is a major source of the bacterial surface lipopolysaccharide (LPS) and other pro-inflammatory molecules and endotoxins. AD patient’s brains contain more frequently pathogenic bacteria and LPS compared to controls (Itzhaki et al., 2004; Fox et al., 2019). LPS was found in the hippocampus and cortex and at higher concentrations in plasma than in healthy controls (Zhao et al., 2017; Kowalski and Mulak, 2019). In addition, LPS co-localizes with Aβ_1–40/42_ in amyloid plaques and around blood vessels (Zhan et al., 2016). Bacterial LPS can bind to microglial receptors (TLR2, TLR4 and CD14) and trigger an inflammatory response. In a recent study LPS was able to strongly activate the NF-kB (p50/p60) complex, an important initiator for neuroinflammatory processes occurring in AD (Lukiw, 2016; Lin et al., 2018).

The most common form of AD typically affects elderly people and aging is associated with significant changes in GM composition. These age-related changes are mainly due to modifications in life-style, diet and a chronic low-grade inflammatory state called “inflammaging” (Santoro et al., 2018). GM in the elderly is reduced in diversity and stability and is less resistant to environmental perturbations such as stress and antibiotics (Biagi et al., 2010). Therefore, it is more vulnerable to the invasion of opportunistic species and to clinically important changes in microbial composition. It has been shown that implantation of aged GM in young GF mice induced inflammaging. In addition, the aged GM promoted small intestine inflammation in implanted GF mice and weakened the intestinal barrier making possible the infiltration of inflammatory bacterial components into the circulation. An increase in systemic T cell activation was also observed (Fransen et al., 2017). In humans, there is an age-related decline in immune system function (Fulop et al., 2018), that could make gut dysbiosis more relevant in triggering low-grade systemic inflammation.

A recent study in mice lacking PINK1^−/−^, which has a key role in adaptive immunity by repressing presentation of mitochondrial antigens, suggests that specific deficits in the immune system function could make intestinal infection a risk factor for neurodegeneration. In these mice, gut infection triggered an autoimmune mechanism involving the mitochondria specific CD8^+^ cells, which are toxic for both peripheral and central neurons. These events led to the degeneration of dopaminergic neurons and motor symptoms typical of PD (Matheoud et al., 2019).

In animal models, dysbiosis increases gut permeability and promotes inflammation and macrophage dysfunction (Thevaranjan et al., 2018). There is evidence of BBB damage and accumulation of blood-derived products in AD brains (Kowalski and Mulak, 2019). The passage of harmful agents from the gut to the brain is still not adequately explained, but compromised the integrity of epithelial barriers might play a role (Sochocka et al., 2019).

Several gut bacterial species such as *E. coli, Salmonella* and *Citrobacter* produce Aβ (O’Toole et al., 2000; Zhou et al., 2012). Amyloids are common structural components of the extracellular matrix in which bacterial cells stay close to each other. Exposure to microbial amyloids might trigger amyloid misfolding in the brain. Increasing evidence supports the idea that the formation and propagation of Aβ seeds is a prion-like mechanism (Walker et al., 2016). However, it is still not clear how bacterial amyloids could gain access to the brain. One possibility is uptake through specialized epithelial cells of the mucosa-associated lymphoid tissues, then physical interaction between enteric nervous system fibers and parasympathetic neurons of vagusnerve where they could reach the CNS *via* retrograde axonal transport (Kujala et al., 2011; Friedland and Chapman, 2017). The key study supporting the hypotheses that the spread of misfolded proteins from the gut to the brain could occur *via* the vagus nerve comes from the context of α-synuclein propagation in PD (Ulusoy et al., 2015; Breen et al., 2019; Santos et al., 2019).

Soscia et al., in 2010 noticed some interesting similarities in biophysical properties of Aβ and a family of biomolecules called “antimicrobial peptides” (AMPs). AMPs are potent broad spectrum antibiotics and modulators of immune system in the brain and other immune-privileged tissues. Dysregulation of these molecules can lead to neurotoxicity and chronic inflammation (Soscia et al., 2010). This study, followed by few others, confirmed the antimicrobial properties of Aβ and proposed its possible physiological role in brain’s innate immune response to microbes. They advanced the hypotheses that brain infiltration of gut bacteria or their components might stimulate Aβ production and deposition (White et al., 2014; Kumar et al., 2016; Eimer et al., 2018).

**Figure 2:**
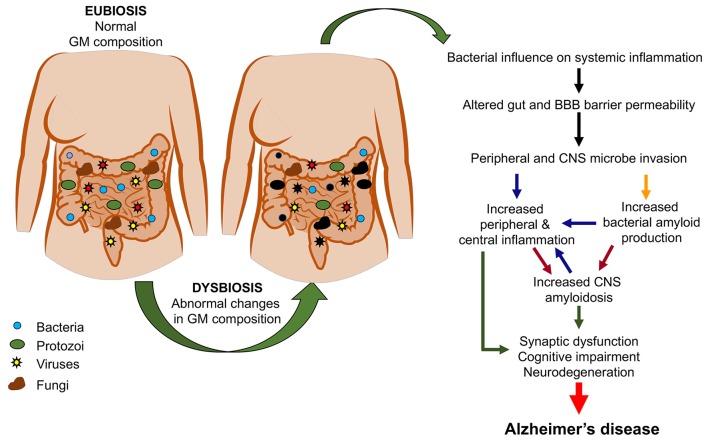
From gut dysbiosis to Alzheimer’s disease (AD). The figure depicts the possible pathological events associated with gut dysbiosis leading to both peripheral and central pathological events which would increase the risk of AD.

Figure 2 summarizes the possible pathological events increasing the risk of AD as a consequence of GM dysbiosis.

## Therapeutic Potential of Microbiota in Neuroinflammation and AD

At present, the existing data on the mechanisms linking GM to neurodegeneration, mostly based on animal studies, are still not sufficient to provide directions for the development of GM-based therapeutic strategies.

Attempts to use probiotics were made mostly in animal models, although there is no evidence that this approach can lead to long-term alterations in GM composition (Akbari et al., 2016; Musa et al., 2017; Plaza-Díaz et al., 2017; Kowalski and Mulak, 2019). In one clinical trial, PD patients were given antibiotics to treat small intestinal bacterial overgrowth, with improvements in gastrointestinal symptoms and motor fluctuations (Fasano et al., 2013). Another strategy for modifying GM composition is fecal transplant, already successful for treating infections of *Clostridium difficile* (Xu et al., 2015), but there are only limited attempts to use it outside gastrointestinal diseases (Evrensel and Ceylan, 2016).

Pre-clinical evidence suggests that microbial metabolism products such as SCFAs could be signaling molecules used by gut microbes to act on the CNS (Erny et al., 2015). Their interaction with the immune system and anti-inflammatory properties make them interesting therapeutic candidates for neurodegenerative disorders. Several strategies for delivery of SCFAs are summarized in Gill et al. (2018).

To date, dietary and lifestyle modifications are the most effective way to produce long-term changes in GM. Some healthy dietary patterns such as Mediterranean, Japanese or FINGER (Finish Geriatric intervention study) diet can positively influence the rates of cognitive decline (Pistollato et al., 2018; Wahl et al., 2019) and also induce significant changes in GM composition.

Among the most significant examples of nutritional intervention with neuroprotective and age-delaying potential is caloric restriction (CR) which can be obtained by reducing the daily caloric intake or by intermittent fasting (Fontana, 2018). CR delays the onset of neurodegeneration in rodents and prevents several hallmarks of brain aging in non-human primates and humans (Pani, 2015). The possible underlying mechanisms are numerous and reviewed in Zullo et al. (2018). Briefly, CR increases levels of neuroprotective factors while decreasing oxidative stress, inflammation, and activity of pro-apoptotic factors (Maalouf et al., 2009). At a molecular level, CR acts on nutrient-sensing pathways through different mechanisms. Notably, it can activate the SIRT1 enzyme (a member of the sirtuin family that regulates gene expression) which downregulates the mammalian target of rapamycin (mTOR), thus suppressing NF-kB-dependent neuroinflammation and inducing autophagy as neuronal self-defense mechanism (Maalouf et al., 2009; Shirooie et al., 2018).

Fasting can induce rapid adaptations in GM composition favoring growth of beneficial and anti-inflammatory microbial phylotypes and lead to significant changes in the SCFA biosynthesis (Zhang et al., 2013; Tanca et al., 2018). GM interacts with several mechanisms of metabolic response to nutrient deprivation. For instance, SIRT1 activation regulates, the GM resulting in lower intestinal inflammation during aging (Wellman et al., 2017). Some substances such as resveratrol, a natural phenol found in grapes and red wine can activate, in alternative to CR, the sirtuine pathway (Kelly, 2010) and positively influence GM. The interaction between resveratrol and GM is bidirectional as gut microbes affect also resveratrol bioavailability (Hu et al., 2019).

CR shares some common metabolic effects with the ketogenic diet (KD) which is high in fat, moderate in proteins and very low in carbohydrate. KD is already used to treat patients with drug resistant epilepsy (Stafstrom and Rho, 2012) and in a few studies have revealed the potential to reduce symptoms of neurodegeneration (Włodarek, 2019). Remarkably, a study using mouse models of drug-resistant epilepsy showed that the KD anti-seizure properties were mediated by microbiota. Depletion of GM with a high dose antibiotic treatment abolished the KD beneficial effects (Olson et al., 2018).

Among other modifiable factors, exercise is considered to promote diversity and enhance beneficial metabolic functions of microbial species in the gut and improve cognitive performance (Ticinesi et al., 2019).

A preventive therapy based on changes in diet and levels of physical activity seems to be the most promising approach for delaying cognitive decline and improving metabolic, neuroendocrine and vascular abnormalities that often precede and likely significantly contribute to cognitive deterioration (Sohn, 2018). A successful preventive strategy must recognize that GM is an important mediator of the effects of diet and exercise on cognitive decline, aging and inflammation. However, additional studies are needed to understand if dietary interventions, such as CR, could be safely recommended to elderly population, which is already at risk of malnutrition and sarcopenia (Sieber, 2019).

One of the present difficulties in tailoring a GM-based therapy is its inter-individual variability in composition and metabolism, but with the rapid advancement in research and diagnostic technologies a new type of personalized medicine might well become possible.

## GM and AD Development: Main Experimental Limitations

Although the study of microbiota-gut-brain axis in recent years has flourished, there are still many obstacles. For instance, a lack of well-defined methodological standards make it hard to compare studies and numerous confounding factors including diet, drugs and concomitant pathologies must be carefully considered in the analysis (Marizzoni et al., 2017). One of the key questions that need to be addressed is whether changes in GM are cause or secondary effects of the disease. At present, GF and antibiotic-treated rodents remain the best available tools for transitioning from observational studies to understanding the cause-effect directionality. However, the translational value of studies of human microbiota in rodent models is limited by obvious differences in diet and microbiota composition.

## Author Contributions

MC and CB wrote the manuscript. GF revised the manuscript.

## Conflict of Interest

The authors declare that the research was conducted in the absence of any commercial or financial relationships that could be construed as a potential conflict of interest.
